# Chloride Ion-Selective Electrode with Solid-Contact Based on Polyaniline Nanofibers and Multiwalled Carbon Nanotubes Nanocomposite

**DOI:** 10.3390/membranes12111150

**Published:** 2022-11-16

**Authors:** Karolina Pietrzak, Klaudia Morawska, Szymon Malinowski, Cecylia Wardak

**Affiliations:** 1 Department of Analytical Chemistry, Institute of Chemical Sciences, Faculty of Chemistry, Maria Curie-Sklodowska University, Maria Curie-Sklodowska Sq. 3, 20-031 Lublin, Poland; 2 Faculty of Civil Engineering and Architecture, Lublin University of Technology, Nadbystrzycka 40, 20-618 Lublin, Poland

**Keywords:** ion-selective electrodes, solid-contact, nanofibers, nanocomposite, potentiometry, chlorides

## Abstract

Use of the nanocomposite of chloride-doped polyaniline nanofibers and multiwalled carbon nanotubes (PANINFs-Cl:MWCNTs) for construction of ion-selective electrodes with solid-contact sensitive to chloride ions has been described. Many types of electrodes were tested, differing in the quantitative and qualitative composition of the layer placed between the electrode material and the ion-selective membrane. Initial tests were carried out, including tests of electrical properties of intermediate solid-contact layers. The obtained ion-selective electrodes had a theoretical slope of the electrode characteristic curve (−61.3 mV dec^−1^), a wide range of linearity (5 × 10^−6^–1 × 10^−1^ mol L^−1^) and good potential stability resistant to changing measurement conditions (redox potential, light, oxygen). The chloride contents in the tap, mineral and river water samples were successfully determined using the electrodes.

## 1. Introduction

Chlorides are widely distributed in the natural environment as salts. They are used in the chemical industry, fertilizer production and food production. It is very important to know the concentration of chloride ions and to monitor them in various types of natural samples and other materials. They are very important, especially in determining the quality of water and its degree of salinity, control of industrial processes or in medicines [1]. It is important to determine their content in food, especially in processed products that are additionally salted in order to preserve them and prevent deterioration. Chlorides, which are naturally present in food products at low levels, can increase significantly during their processing, cooking and seasoning. The chloride concentration in drinking water is, on average, below 50 mg L^−1^. The balance of electrolytes in the body is maintained by regulating total intake and excretion through the kidneys and the gastrointestinal tract. Considering the average chloride excretion from the body, an intake of 3.1 g/day for adults was considered recommended. No toxicity of chlorides was found in adults where metabolism of sodium chloride was working properly without any disturbances [2]. A number of methods have been developed that can be used to determine chloride content in various products and materials, including chronopotentiometry in long-term monitoring of chloride content in cement-based materials [3,4,5], chromatography methods in meat samples [6] or electrochemical methods—in sea water [7], desalted water [8], blood [9], milk [10] and food [11,12].

Among electrochemical techniques, potentiometry distinguishes itself due to its low cost, simplicity and high speed of measurements. This method enables determination of ions in colored and muddy samples, which usually do not require any pretreatment [13,14]. The most popular group of potentiometric sensors are ion-selective electrodes (ISEs), which work by converting the activity of the ion into an electric potential that can be measured [15]. ISEs can be used to determine the content of selected ions in various types of liquid samples (water, drinks and even blood). However, to be considered fully functional and working properly, ISEs should meet a number of conditions. In their case, the key parameter is high selectivity, which makes it possible to determine the main ion concentration in real samples containing other ions [16]. In addition, the stability and reversibility of the potential are crucial so that the sensors can be used to perform measurements repeatedly over a longer period of time and the results obtained can be considered reliable. What is more, the electrical parameters of the electrodes are also important, which can be estimated on the basis of measurements using impedance spectroscopy and chronopotentiometry [17,18]. It is particularly important to develop electrodes with smaller sizes, different shapes and better mechanical resistance so that they can be used for automatic and direct determination of the content of selected ions in an in situ environment even without the need to collect samples and analyze them in the lab [1,19,20]. Such advantages are characteristic of ion-selective electrodes with solid-contact (SCISEs) in which the internal solution has been eliminated. However, to ensure the stability of the potential, solid-contact was used as a transducer, enabling the charge transfer between the solid electrode material and the ion-selective conductive membrane [21]. SCISEs, unlike conventional electrodes with an internal solution, are insensitive to liquid evaporation and changes in sample temperature and pressure and are easier to store and transport [17].

Thus far, research on obtaining various types of ion-selective electrodes sensitive to chloride ions has been described in the scientific literature several times. Depending on the materials and chemicals used, the sensors had different analytical parameters. In the work described by Legin et al., after optimizing the composition of the ion-selective membrane containing tridodecylmethylammonium chloride (TDMACl) as the active substance, a chloride electrode was obtained, characterized by a calibration slope of −48.4 mV dec^−1^ [22]. In order to analyze chlorides in pharmaceutical solutions, indium(III) octaethyl-porphyrin was used as an ionophore and electrodes with a super-Nernstian slope were obtained [23]. Electrodes with a slope of −55.0 mV dec^−1^ and linearity of the concentration range of 1 × 10^−3^–1 × 10^−1^ mol L^−1^ were obtained in studies by Kim et al. The effect of a number of interfering ions that may be present in the sample solution was investigated, including CN^−^, Br^−^, ClO_4_^−^, SCN^−^, acetate, hydrogen carbonate, lactate, citrate and salicylate ions [24]. Graphitic carbon nitride/silver chloride composite was also used for construction of chloride carbon paste electrodes to generate sensors with a linearity range of 1 × 10^−6^–1 × 10^−1^ mol L^−1^ and a slope of −55.4 mV dec^−1^. In their case, the interfering ions were CN^−^, I^−^, Br^−^. They were then used to test samples of river water, sea water and drinking water with satisfactory results [25]. A wide range of linearity of the calibration curve of 5 × 10^−8^–1 × 10^−1^ mol L^−1^ and a low detection limit were achieved for electrodes in which the anionic receptor 2-(1-H-imidazo [4,5-f][1,10]phenanthroline-2-yl)-6-methoxyphenol (HIPM) was used as the main membrane component. These electrodes were also successfully used to determine chloride ions in water, although the pH range declared by the authors in which the electrodes can be used was only 6.5–8.0 [26]. Sensors that can work in a wide range of pH changes were obtained for the purpose of research on corrosive processes. For the glass capillary microelectrodes constructed for this purpose, a slope of −58.7 mV dec^−1^ was achieved in the range 1 × 10^−4^–1 × 10^−1^ mol L^−1^ [27]. Research on a potentiometric chip-based flow system for simultaneous determination of chlorides, fluorides, pH and redox potential in water samples [28] and an MIP-202-catalyst-integrated chloride sensor for detection of a sulfur mustard stimulant [29] has also been described. However, a review of the literature in the field of chloride electrodes shows that there is still a need for research on development of electrodes showing good analytical parameters.

Regarding SCISEs, the properties of the solid-contact material have a significant impact on the parameters of the electrodes. Substances that can be successfully used as solid-contacts in ISEs should meet a number of requirements. They should have electric and ionic conductivity, reversibility and be sufficiently chemically stable not to undergo undesirable reactions during this process. In addition, they should be sufficiently hydrophobic to prevent formation of a water layer between the solid electrode material and the ion-selective membrane and have high bulk capacitance to ensure stable potential [30]. Conductive polymers were the first to be used as SC, such as poly(pyrrole) [31], poly(3-octylthiophene) [32] or polyaniline [33]. In recent years, nanomaterials, especially carbon-based nanomaterials, have gained great popularity in potentiometry. Properties such as high charge transfer, remarkable electrical capacities and good hydrophobicity make them ideal for use as transducer elements in potentiometric sensors [34]. Thus far, many types of nanomaterials (e.g., nanotubes, nanofibers, nanorods, nanowires, nanoparticles, nanocomposites and others) were used for this purpose [30]. From nanoparticles, scientists have described research on use of mainly metal nanoparticles: gold [35], silver [36], platinum [37] or metal oxide nanoparticles [38]. Recently, we reported successful use of polyaniline nanofibers doped with chloride and nitrate ions as solid-contact in nitrate ion-selective electrodes [39]. Polyaniline nanofibers (PANINFs) combine the unique properties of nanomaterials with the mixed ionic and electronic conductivity of conductive polymers. PANINFs and multiwalled carbon nanotubes (MWCNTs) form a nanocomposite with better electrical properties than its individual components (lower resistance and higher capacitance). It seems that such a nanocomposite is a good candidate as solid-contact for preparation of potentiometric sensors. In combination with a polymer membrane containing a highly selective ionophore, it provides hope for obtaining electrodes with good analytical and operational parameters. This work reports the study of electric properties of PANINFs and MWCNTs nanocomposite and its first usage as solid-contact in electrodes sensitive to chloride ions.

## 2. Materials and Methods

### 2.1. Apparatus

For potentiometric research, a cell consisting of the tested ion-selective electrodes (suitably modified glassy carbon electrodes (GCEs)) and a silver/silver chloride reference electrode with a double junction system (6.0750.100, Metrohm, Herisau, Switzerland) was used. The electromotive force (EMF) measurements were made at room temperature in mixed solutions using a magnetic stirrer. A 16-channel data acquisition system (Lawson Labs. Inc., Malvern, PA, USA) connected to a computer with appropriate software was used for data collection.

Electrochemical impedance spectroscopy and chronopotentiometry measurements were carried out for a 3-electrode system in which the tested electrode (GCE covered by the studied nanomaterial or ion-selective electrode) was the working electrode, Ag/AgCl (6.0733.100, Metrohm, Herisau, Switzerland)—reference electrode and GC rod 2 mm/65 mm (Metrohm, Herisau, Switzerland)—auxiliary electrode. All measurements were conducted in a NaCl solution with a concentration of 10^−1^ mol L^−1^. The impedance spectra were recorded in the frequency range 0.1–100 kHz and 0.01–100 kHz (for the intermediate layers and ion-selective electrodes, respectively) at the open circuit potential with an amplitude of 10 mV. In chronopotentiometry measurements, a constant current of +1 µA and +100 nA (for the intermediate layers and ion-selective electrodes, respectively) was applied on the working electrode for 60 s, followed by a current of −1 µA and −100 nA for next 60 s, with simultaneous recording of the electrode potential. The AUTOLAB electrochemical analyzer (Eco Chemie, Utrecht, The Netherlands) and NOVA 2.1 software were used to perform the above measurements, collect them and adjust the electric circuit to the obtained impedance spectra.

The images of PANI/MWCNTs nanocomposite structure were recorded using a high-resolution scanning electron microscope Quanta 3D FEG (FEI Hillsboro, Hillsboro, OR, USA).

### 2.2. Reagents

Chemical substances used for synthesis of aniline nanofibers, aniline monomer, hydrochloric acid (HCl), ammonium persulfate (APS) and tetrahydrofuran (THF), were purchased from Chempur (Piekary Slaskie, Poland). Polyaniline nanofibers doped with Cl^-^ ion-synthesized following the procedure described in the publication [39]. Substances necessary for preparation of the membrane mixture were purchased from Sigma-Aldrich (Saint Louis, MO, USA) (chloride ionophore III-selectophore, tridodecylmethylammonium chloride (TDMACl) and high-molecular-weight poly(vinyl) chloride) (PVC)) and Fluka (Buchs, Switzerland) (bis(2-ethylhexyl) sebacate (DOS)). Sulfuric acid and sodium hydroxide used to measure the dependence of the electrode potential on changes in pH were obtained from Chempur, while the salts of iron(II) (Na_4_Fe(CN)_6_ × 10H_2_O) and iron(III) (K_3_Fe(CN)_6_) were used to prepare the solutions differing in redox potential, respectively, from Alfa Aesar (Haverhill, MA, USA) and PPH (Polish Chemical Reagents, Gliwice, Poland). Other substances, such as inorganic salts, used to prepare the solution of the main ion (NaCl) and sodium salts of selected interfering anions (NaH_2_PO_4_, CH_3_COONa, Na_2_CO_3_, NaNO_3_, NaNO_2_, Na_2_SO_4_, NaF, NaBr) were purchased from Fluka. Salts of the highest purity available (pure pro analysis) and freshly deionized water were used to prepare all solutions.

### 2.3. Preparation of Intermediate Solid-Contact Layers

Both the nanofibers (PANINFs-Cl) and the nanotubes (MWCNTs) as well as different nanocomposites made of them were used as intermediate layers of solid-contact in the ion-selective electrodes. Nanocomposites with a weight ratio of PANINFs-Cl:MWCNTs equal to 1:2, 1:1 and 2:1 were obtained by mixing the weighed components in THF, thoroughly homogenizing the mixture in an ultrasonic bath for one hour. Each time, the mass of components equal to 0.01 g was weighed on an analytical balance, to which 1 mL of THF was then added to obtain the initial concentration of components equal to 10 mg mL^−1^. Then, in order to perform preliminary tests involving the examination of electrical parameters of materials and their nanocomposites, 10 µL volumes of their homogenized suspensions were spotted onto properly cleaned and dried glassy carbon electrode surfaces (GCE) and were allowed to evaporate the solvent.

### 2.4. Preparation of the Ion-Selective Membrane

The ion-selective membrane mixture was prepared by weighing its components on an analytical balance and thoroughly mixing it with THF using an ultrasonic bath. Ingredients with a total weight of 0.3 g were prepared and then added with 3 mL of THF. The qualitative and quantitative composition of the membrane was as follows: 2.0% chloride ionophore III, 1.2% TDMACl, 33% PVC and 63.8% DOS (as recommended by the producer [40]). After all the membrane components were homogenized completely in the organic solvent, the membrane was ready to be applied to the properly prepared electrode surface.

### 2.5. Preparation of Solid-Contact Ion-Selective Electrodes

For the construction of ion-selective electrodes, glassy carbon electrodes (GCEs) with a diameter of 0.3 cm were used. The surface of the electrodes was properly prepared before the application of successive layers. They were cleaned with sandpaper, grain sizes 2500 and 5000, then polished with alumina powder (0.3 µm size), wetted with distilled water and rinsed thoroughly. An ultrasonic bath was used to get rid of the residual alumina. Finally, the electrodes were rinsed again abundantly with distilled water, then with an organic solvent, THF, which was also used to prepare the membrane mixture. The electrodes were allowed to dry. Then, 10 µL of nanomaterials dispersed in THF were dropped on each electrode to thoroughly coat the solid-contact interlayer (except for the electrodes intended to act as basic electrodes containing the ion-selective membrane itself placed directly on the electrode material). Next day, the ion-selective membrane was dropped on every electrode—3 layers of 30 µL, each time allowing the solvent to evaporate for 30 min. The electrodes with the spotted ion-selective membrane were allowed to dry overnight. Then, all electrodes were stored immersed in a conditioning solution—10^−3^ mol L^−1^ NaCl in a dark and dry place.

## 3. Results and Discussion

### 3.1. Characterization of Solid-Contact Materials

#### 3.1.1. SEM Images

**Figure 1:**
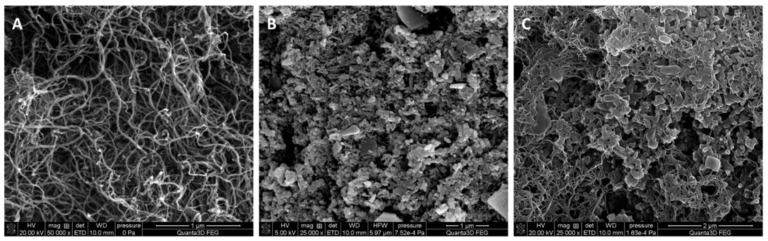
SEM images obtained for the layers of (**A**) MWCNTs, (**B**) PANINFs-Cl, (**C**) (2:1)PANINFs-Cl:MWCNTs nanocomposite.

In order to compare the structure of the used types of solid-contact, they were studied by scanning electron microscopy technique. The scanning electron micrographs shown in Figure 1 clearly confirm the difference in the structure of the MWCNTs (A), PANINFs-Cl (B) and the nanocomposite (C) obtained from both of these components. In image 1C, polyaniline nanofibers entwined by carbon nanotubes can be observed.

#### 3.1.2. Chronopotentiometric Tests of the Intermediate Layer

**Figure 2:**
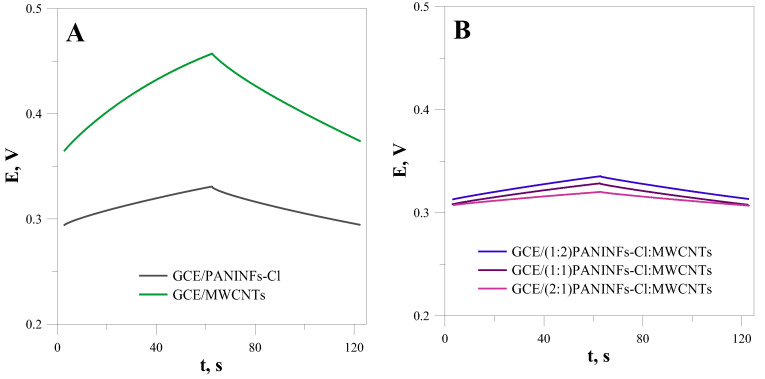
Chronopotentiometric curves obtained for the layers of (**A**) PANINFs-Cl and MWCNTs; (**B**) nanocomposites PANINFs-Cl:MWCNTs.

The next step of the study was to examine the electric properties of the studied nanomaterials using chronopotentiometry (CP) and electrochemical impedance spectroscopy (EIS). First, the electric parameters of the obtained layers were determined by the chronopotentiometry method in a NaCl solution of 10^−1^ mol L^−1^. The electric capacity of the tested materials was so large that it was necessary to use a current of 1 µA for measurements involving only the intermediate layers (without the spotted membranes). The results obtained for the GCE modified by PANINFs, MWCNTs and their nanocomposites are presented in Figure 2. Based on the course of the chronopotentiometric curves and formulas: R = E/i; drift = ∆E/∆t = i/C (where E–potential change, i–applied current, t–time change), the electric capacitance (C) and resistance of the electrode (R) were determined (Table 1) [18]. As all the electrodes differed only in the type of nanomaterial covering the GCE, the observed differences resulted from their different properties.

In the case of the nanocomposite, a synergistic effect was observed. It was found that all the electrodes obtained from the nanocomposites showed higher electric capacitance and lower resistance than the electrodes obtained only from PANINFs-Cl or MWCNTs. The nanocomposite obtained from PANINFs-Cl and MWCNTs with a 2:1 weight ratio showed the most favorable electric properties.

#### 3.1.3. Initial Electrochemical Impedance Spectroscopy Tests of the Intermediate Layer

**Figure 3:**
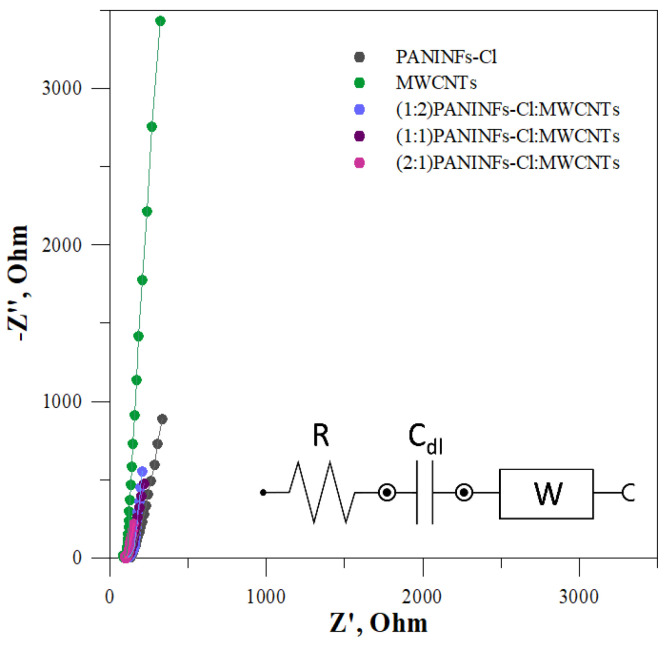
Impedance spectra for all solid-contact layers measured in 10^−1^ mol L^−1^ NaCl with an equivalent electrical circuit (solid lines represent data fits; the error of the fits χ^2^ is given in Table 2).

The intermediate solid-contact layers were also tested by EIS. The impedance spectra were recorded in the frequency range 0.1–100 kHz at the open circuit potential, with an amplitude of 10 mV. The obtained impedance spectra and the electrical circuit that was matched for the electrodes are shown in Figure 3. The electrical circuit consists of the uncompensated series resistance (R), mainly electrolyte resistance, the Warburg impedance (W) connected to the ion transport in the solid-contact layer and double layer capacitance (C_dl_) [41]. The determined data are presented in Table 2, where it is evident that the studied nanomaterials show different capacities. In each case, the nanocomposite had a greater double layer capacitance C_dl_ than its constituent components, i.e., PANINFs-Cl and MWCNTs. The nanocomposite (2:1)PANINFs-Cl:MWCNTs was characterized by the largest value of C_dl_ = 7.01 mF, which was over ten times greater than the C_dl_ value obtained for MWCNTs (0.59 mF) and more than three times greater than the C_dl_ value obtained for PANINFs-Cl (2.10 mF).

### 3.2. Characterization of Ion-Selective Electrodes

#### 3.2.1. Electrical Parameters of Ion-Selective Electrodes

**Figure 4:**
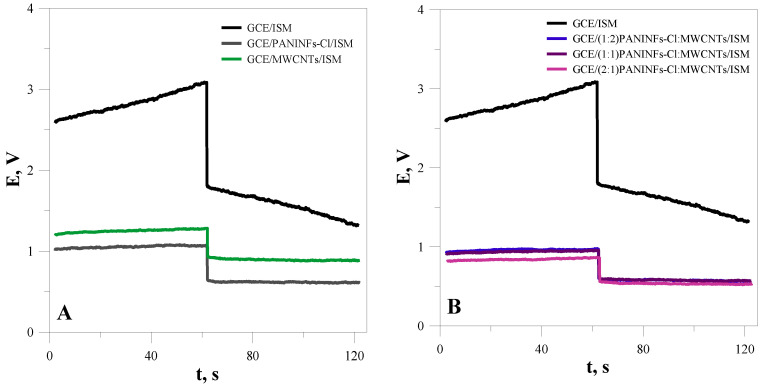
Chronopotentiometric curves obtained for the electrodes: (**A**) unmodified GCE/ISM and with intermediate layers of nanofibers GCE/PANINFs-Cl/ISM or nanotubes GCE/MWCNTs/ISM; (**B**) unmodified GCE/ISM and with intermediate layers of nanocomposites.

In order to check the extent to which the properties of the intermediate layer of nanomaterials affect the electrical parameters of ion-selective electrodes, complete sensors with an intermediate layer and an ion-selective membrane were also tested using the CP and EIS methods. For prepared electrodes that, in addition to the intermediate layer, also had a membrane layer, a current of 100 nA was selected for chronopotentiometric measurements. Figure 4 shows the chronopotentiometric curves obtained for the electrodes with intermediate layers and for the unmodified electrode (GCE/ISM). As was expected, the electrodes with the intermediate layer exhibited better electric parameters (higher capacitance and lower total resistance) than the simple coated disc electrode. Due to this, the modified electrodes showed reduced potential drifts upon galvanostatic polarization compared with the unmodified electrode (Table 3). This effect was the largest for the electrode based on the nanocomposite (2:1)PANINFs-Cl:MWCNTs/ISM. The potential stabilizing effect is connected with the presence of the nanomaterial layer that was placed between the ion-sensitive membrane and the inner electrode and depends on its capacitance.

**Figure 5:**
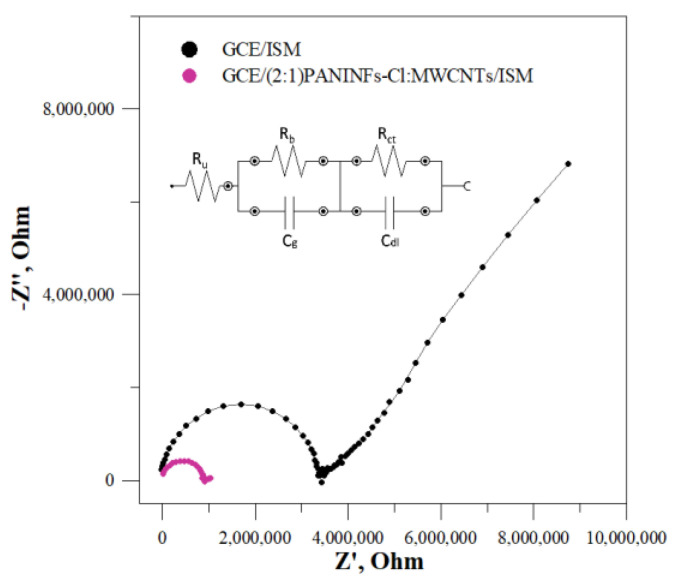
Impedance spectra for GCE/ISM and GCE/(2:1)PANINFs-Cl:MWCNTs/ISM measured in 10^−1^ mol L^−1^ NaCl and equivalent circuit (solid lines represent data fits, the error of the fits χ^2^ was 0.056 for GCE/ISM and 0.012 for GCE/(2:1)PANINFs-Cl:MWCNTs/ISM).

The beneficial effect of the presence of the interfacial layer on the electrodes’ electric parameters was confirmed by EIS study. Electrochemical impedance spectroscopy is a very useful technique to study electrochemical processes. In relation to ion-selective electrodes, it allows for the determination of, inter alia, charge transfer resistance, which provides us information about the efficiency of the intermediate layer. Impedance spectra were recorded at an open circuit potential with an amplitude of 10 mV, while the frequency range was 0.01–100 kHz. The obtained impedance spectra for the best electrode GCE/(2:1)PANINFs-Cl:MWCNTs/ISM and the unmodified electrode GCE/ISM are shown in Figure 5. The fitted equivalent circuit is presented in the insert. In the case of the electrode without the intermediate layer, a large semicircle in the high frequency region and a huge partial semicircle in the low frequency region are observed. Both parts of the impedance spectra are dramatically diminished in the case of the nanocomposite ((2:1)PANINFs-Cl:MWCNTs)-modified electrode. The high-frequency semicircle can be attributed to the bulk resistance (R_b_) and geometric capacitance (C_g_) of the ISM, while the low-frequency part of the semicircle can be connected to the charge transfer resistance (R_ct_) in parallel with double layer capacitance (C_dl_) at the interface between the polymeric membrane and the inner GC electrode. The obtained impedance spectra were fitted to the equivalent circuit shown in the insert of Figure 5 and the particular electric parameters of the electrodes were determined. The bulk membrane resistance R_b_ decreased from 3.4 MΩ for the unmodified GCE/ISM to 0.91 MΩ for the GCE/(2:1)PANINFs-Cl:MWCNTs/ISM, respectively. The same effect but to a much greater extent was observed for the charge transfer resistance R_ct_, which decreased from 29.7 MΩ for the unmodified CGE/ISM to 0.14 MΩ for the nanocomposite-based electrode, respectively. Concurrently, the low frequency layer capacitance (C_dl_) increased drastically. It was 7.3 pF for the unmodified GCE/ISM, much smaller than the value obtained for the nanocomposite-based electrode, whose C_dl_ was 1.4 µF. These results confirm that the studied nanocomposite (2:1)PANINFs-Cl:MWCNTs significantly facilitates the diffusion processes and charge transport at the membrane/GCE interface and is a promising material for intermediate layers in solid-contact ISEs.

#### 3.2.2. Potentiometric Response

**Figure 6:**
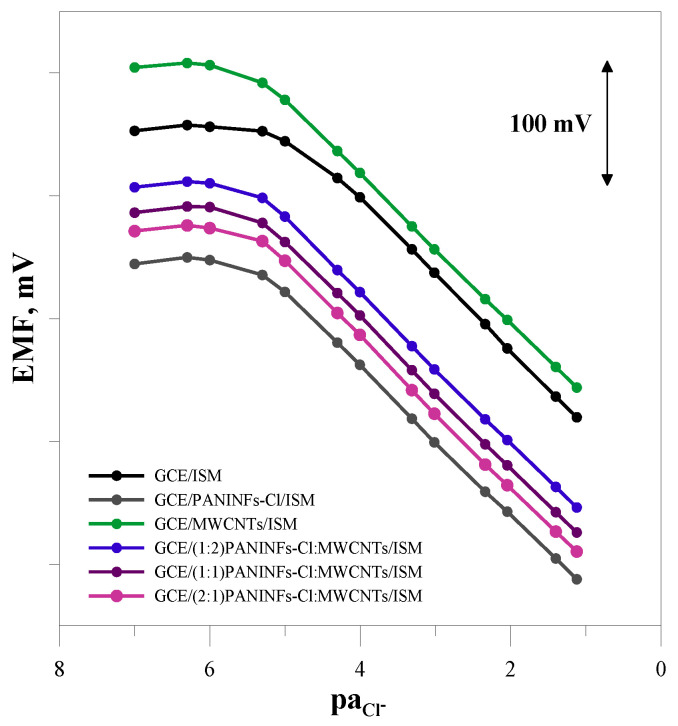
Potentiometric calibration plots recorded for the tested electrodes in NaCl solutions (concentration range 10^−7^–10^−1^ mol L^−1^).

The potentiometric response of the sensors was tested in NaCl solutions in the concentration range of 10^−7^–10^−1^ mol L^−1^ (every half unit). The electromotive force (EMF) of the cell was measured in mixed solutions. Measurements to obtain calibration curves of the tested electrodes were performed twice a week for a period of 2 months. The slope of the calibration curve as well as the range of its linearity and the limit of detection were checked. The E^0^ value was also determined each time by extrapolating the linear segment of the response function to paCl^−^ = 0. The exemplary calibration curves obtained one week after preparation of individual sensors (the graph of the potential versus the negative logarithm of the activity of chloride ions in the solution) are shown in Figure 6. As can be seen, all the obtained electrodes were sensitive to chloride ions and showed a characteristic slope close to the theoretical value. The electrode response differed in the measuring range and limit of detection. The unmodified electrode had the shortest linear range of the calibration curve (5 × 10^−5^–1 × 10^−1^ mol L^−1^) and the highest limit of detection, which was 6.3 × 10^−6^ mol L^−1^. The modified electrodes, regardless of the type of the intermediate layer, showed a similar potentiometric response. Compared to the unmodified electrode, their measurement range was wider by one unit pa, and the detection limit was much lower (Table 4).

The electrode performance changed over time but to a different extent. Over the two-month period, all the electrodes kept a very good slope of the calibration curve. In the case of the unmodified electrode, the measurement range shortened and the detection limit increased by half an order. On the other hand, the electrodes with an intermediate layer (except GCE/PANINFs-Cl/ISM) showed an unchanged measuring range and a slightly worse detection limit.

The greatest differences were observed in the value of E^0^. This parameter is a measure of the long-term stability of the potential, and changes in the value of E^0^ are a source of measurement errors. An electrode characterized by a stable E^0^ value does not require control calibrations and allows correct results of determinations to be obtained. The average values of E^0^ determined from successive measurements and the standard deviation from the mean value are provided in Table 4, where the modified electrodes showed much better stability of the E^0^ potential compared to the unmodified electrode. The improvement in long-term stability (E^0^ potential change) is related to the electric capacitance of the intermediate layer and is the greatest for the electrode GCE/(2:1)PANINFs-Cl:MWCNTs/ISM with a nanocomposite layer characterized by the best electrical parameters.

#### 3.2.3. Short-Term Stability and Reversibility of the Electrode Potential

**Figure 7:**
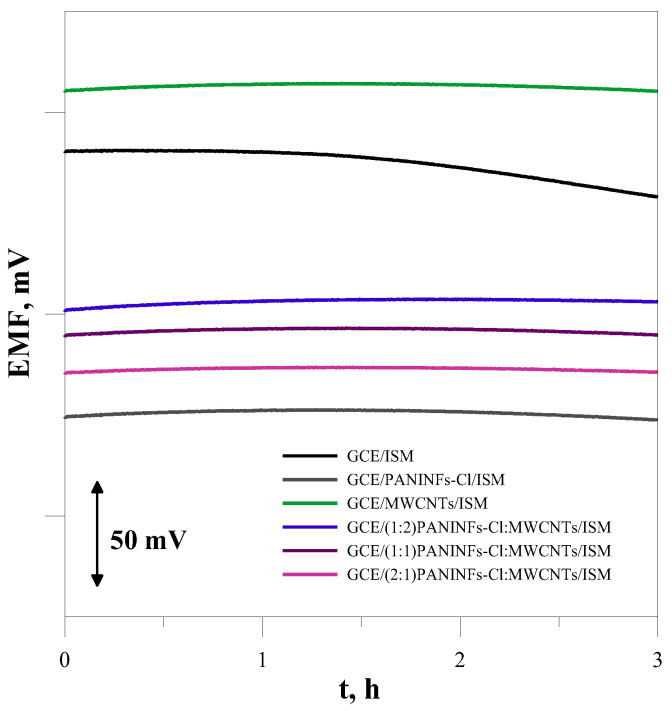
The short-term potential stability measured in 10^−3^ mol L^−1^ NaCl.

The short-term stability of the electrode potential was measured in a 1 × 10^−3^ mol L^−1^ NaCl solution for 3 h. From the recorded potential change in time (Figure 7), the potential drift under zero current conditions was determined as (ΔE/Δt) and the calculated values are provided in the last column of Table 5. As was expected, all the modified electrodes show very good potential stability. They exhibited potential drift much smaller than the electrode without an intermediate layer. It is within this time (Figure 7).

The reversibility of the potential of the tested electrodes was also measured. For this purpose, the solutions of the main ion salt (NaCl) with a concentration of 1 × 10^−4^ and 1 × 10^−3^ mol L^−1^ were changed every 10 min and the obtained potential values were read. The operation was repeated five times for each of the solutions, then the mean potentials and standard deviation were calculated; the results obtained are summarized in Table 5. A quantitative measure of the reversibility of the electrode potential is the value of the standard deviation from the mean value of the potential measured in successive tests in a solution with a given concentration. From the analysis of the data in Table 5, it can be concluded that, in each case, the introduction of the intermediate layer causes a significant improvement in the reversibility of the potential, the effect being the greatest for (2:1)PANINFs-Cl:MWCNTs-nanocomposite-modified electrodes.

#### 3.2.4. Selectivity

The selectivity of the tested electrodes was estimated by determining the selectivity coefficients by the method of separate solutions. The variant of this method proposed by Bakker [42] was applied, in which the values of the selectivity coefficients are calculated from the equation logK^pot^_Cl/X_ = –(E_X_−E_Cl_)/S, where E_X_ is electrode potential in the interfering ion solution with activity a_X_ = 1; E_Cl_ is electrode potential in the chloride solution with activity a_Cl_ = 1 and S is the slope of the electrode response in chloride solution. 

Selectivity coefficients for various anions were estimated, including H_2_PO_4_^−^, CH_3_COO^−^, HCO_3_^−^, NO_3_^−^, NO_2_^−^, SO_4_^2−^, F^−^ and Br^−^ ions. The tested electrodes did not differ significantly in terms of selectivity. The obtained selectivity coefficients had similar values for all the modified and unmodified electrodes. This proves that the type of solid-contact material does not affect the selectivity of the electrode, which is determined by the composition of the polymeric membrane (mainly ionophore). In this case, all the electrodes had the same membrane, and, therefore, they showed similar values of the selectivity coefficients. The determined values of logK^pot^_Cl/X_ for the GCE/(2:1)PANINFs-Cl:MWCNTs/ISM decreased in the order −1.6, −4.6, −4.8, −5.0, −5.1, −5.6, −6.4 and −6.6 for Br^−^, NO_3_^−^, HCO_3_^−^, NO_2_^−^, SO_4_^2−^, H_2_PO_4_^−^, CH_3_COO^−^ and F^−^, respectively. Such values indicate very good selectivity of the electrodes, which makes them suitable for determination of chloride ions in various samples.

#### 3.2.5. pH Range

**Figure 8:**
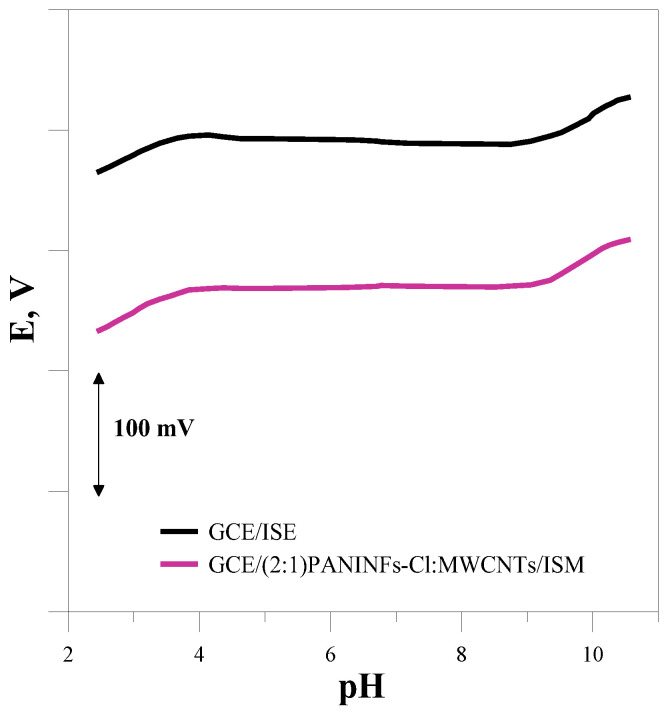
Dependence of electrode potential on pH.

The measurements were performed to determine the pH range in which the tested electrodes can be successfully used to determine the concentration of chloride ions. The electrode potential was measured in solutions of the main ion with a concentration of 10^−3^ mol L^−1^ with different pH values. Sulfuric acid and sodium base were used to obtain the appropriate pH of the solutions. All the electrodes showed a stable potential over a similar pH range of around 4–9. Since the same membrane mixture was used to construct the electrodes, it was found that the use of different materials as an intermediate layer of solid-contact is irrelevant to the pH range in which the sensors can be used. Figure 8 shows the dependence of the electrode potential on pH for the unmodified GCE/ISM and nanocomposite-based electrode GCE/(2:1)PANINFs-Cl:MWCNTs/ISM.

#### 3.2.6. Redox Sensitivity

**Figure 9:**
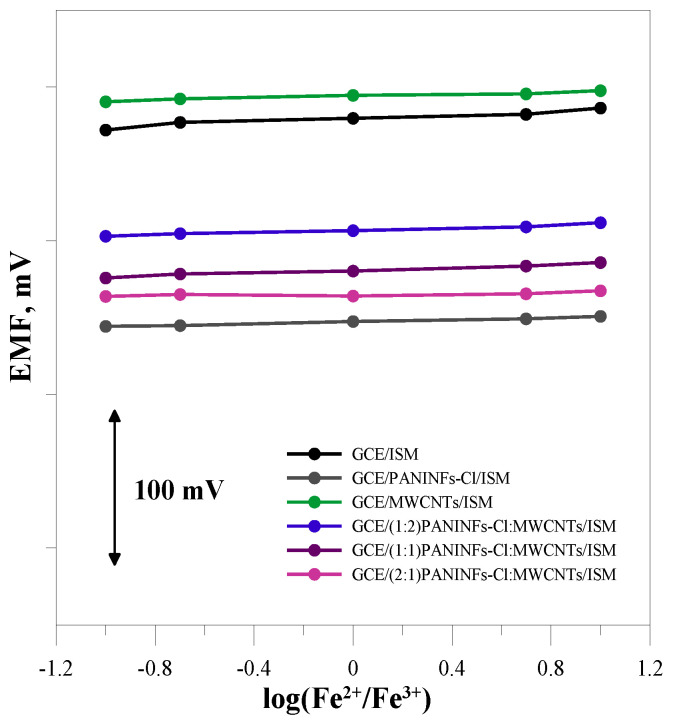
Redox sensitivity for tested electrodes.

Potentiometric measurements were performed in solutions with different redox potential in order to check the redox sensitivity of the tested electrodes (Figure 9). Solutions with a concentration of 10^−2^ mol L^−1^ NaCl were used, which contained Fe(CN)_6_^3−^ and Fe(CN)_6_^4−^ redox coupled with log([Fe^2+^]/[Fe^3+^]) equal to −1, −0.7, 0, 0.7 and 1. The potential of the electrodes measured in solutions with different redox potential does not change significantly. It can, therefore, be considered that they work properly regardless of the redox potential of the sample.

#### 3.2.7. Sensitivity to Light and Oxygen

**Figure 10:**
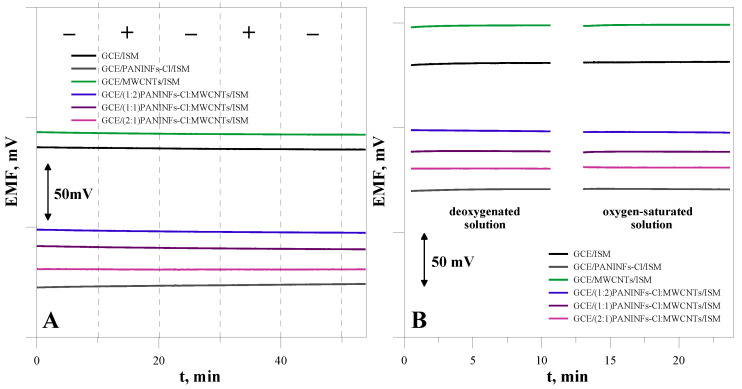
The effect of (**A**) light, (**B**) O_2_ on the electrode potential.

It is known that SCISEs based on conducting polymers can be sensitive to light and the presence of gases, such as oxygen or carbon dioxide [43,44]. Therefore, the influence of the presence of light and gases on the stability of the electrode potential was investigated. The potential was measured in a solution of the main ion with a concentration of 10^−1^ mol L^−1^. To examine the effect of the presence of gases on changes in the electrode potential, measurements were performed in solutions saturated with gases, alternately with solutions deoxygenated by passing nitrogen through the solution for one hour. The obtained dependence of the potential on time under changing lighting conditions is presented in Figure 10A, while variable conditions regarding the presence of oxygen in the sample solution are in Figure 10B. As can be seen in these figures, all the tested electrodes were resistant to changes in light and the presence of gases (O_2_, CO_2_).

### 3.3. Determination of Chlorides in Real Samples Using the Proposed Electrode

In order to check the effectiveness of using the electrodes in the study of real samples, determination of chloride concentrations in the samples of drinking water (tap water and mineral water) and river water was performed using the GCE/(2:1)PANINFs-Cl:MWCNTs/ISM. The method of classical quantitative analysis was used as a comparative method: determination of chlorides by Mohr’s method. Classical quantitative analysis methods in comparison to instrumental methods have the following characteristics: if it is necessary to observe the visual endpoint of titration, i.e., the change in color of the solution from one drop of the titrant, this method depends to a large extent on the person performing the analysis. In addition, it is necessary to have the right type of glass and access to appropriate reagents and indicators, the addition of which enables detection of the endpoint of titration (here, potassium chromate) and solutions of substances acting as a titrant, the titer of which has been correctly and accurately determined. In addition, such analysis takes much more time, and, if the concentration range of the substance in the sample is not known, it may be necessary to dilute the sample and/or the titrant appropriately to fit the titrant volume not exceeding the burette volume, and for greater accuracy of the read volume—preferably within the range 20–80% of its volume. In the case of instrumental methods, and more precisely in potentiometry using ISEs, the measurements are much faster, less complicated and rely to a much lesser extent on the senses of the person. In addition, depending on the type of electrodes, the range of linearity of their calibration curves covers several levels of concentration, so it is possible to determine samples that differ significantly in the content of the tested ions.

In the case of potentiometric measurements, the only step in the preparation of the sample was the addition of sodium acetate as an ionic strength buffer, each time obtaining its concentration of 10^−2^ mol L^−1^. In the same environment, a calibration curve for the tested electrodes was previously prepared. Chloride concentration in water samples was estimated by the standard addition method for each sample and electrode in three replicates. In the case of classical quantitative analysis, the titration was performed using of silver nitrate titrant in the presence of a color indicator of potassium chromate. It is worth noting that, in the case of the potentiometric method, the required sample volume is much smaller than in the case of classical analysis. The obtained mean results, including standard deviations, are summarized in Table 6.

## 4. Conclusions

The modified electrodes were characterized by a wider measuring range and a lower detection limit compared to the unmodified electrode without a solid-contact layer. The obtained sensors had a high slope of the calibration curve, a wide measuring range, a very good potential stability, and a fast response time. This effect was the largest in the case of the PANINFs-MWCNTs nanocomposite-based electrode (in particular, for a 2:1 ratio of PANINFs-Cl:MWCNTs). Moreover, they were insensitive to change in redox potential, as well as light and oxygen, which is important from the practical point of view. The obtained electrodes were successfully used to test water samples (tap water, mineral water and river water). Due to their wide measuring range and very good selectivity, they can be used, for example, to control the efficiency of water desalination.

Chloride ion-selective electrodes are available commercially, including from the companies ELMETRON, HACH, MERA, VERNIER and THERMOFISHER (Table 7). The obtained electrodes with a composite interlayer of (2:1)PANINFs-Cl:MWCNTs are characterized by a wider range of linearity compared to the vast majority of them and have better selectivity and a fast sensor response. Most manufacturers declare a very wide pH range (2–11) in which the electrodes can be used; for testing natural water samples, usually a pH range of 4–9 should be sufficient. In addition, it is possible to add a buffer to the sample, ensuring an appropriate pH of the solution. Whenever the selectivity of chloride electrodes over other ions is tested, a list of interfering ions is available, confirming that there is no ideally selective chloride electrode available. The price of the sensors is also an important aspect as crystalline chloride electrodes are usually quite expensive.

## Figures and Tables

**Table 1:** Estimated values of electric capacitance (C) and resistance (R) for the tested solid-contact layers determined by chronopotentiometry.

Layer Material	C, mF	R, kΩ
PANINFs-Cl	1.82	0.64
MWCNTs	0.68	0.61
(1:2)PANINFs-Cl:MWCNTs	2.70	0.37
(1:1)PANINFs-Cl:MWCNTs	3.01	0.31
(2:1)PANINFs-Cl:MWCNTs	7.16	0.21

**Table 2:** Electrical parameters for the tested electrodes determined by EIS.

Layer Material	R [Ω]	C_dl_ (mF)	W (mOhm × s^(1/2)^)	χ^2^
PANINFs-Cl	123.0	2.10	4.78	0.089
MWCNTs	95.0	0.59	5.76	0.092
(1:2)PANINFs-Cl:MWCNTs	108.0	3.12	7.95	0.096
(1:1)PANINFs-Cl:MWCNTs	103.0	3.40	8.59	0.054
(2:1)PANINFs-Cl:MWCNTs	101.0	7.01	13.6	0.058

**Table 3:** Estimated values of electric capacitance (C) and resistance (R) for the tested electrodes determined by chronopotentiometry.

Electrode	C, mF	ΔE/Δt, mV s^−1^	R, MΩ
GCE/ISM	0.013	7.7	8.01
GCE/PANINFs-Cl/ISM	0.12	0.81	4.11
GCE/MWCNTs/ISM	0.10	0.96	3.49
GCE/(1:2)PANINFs-Cl:MWCNTs/ISM	0.28	0.36	3.66
GCE/(1:1)PANINFs-Cl:MWCNTs/ISM	0.26	0.34	3.48
GCE/(2:1)PANINFs-Cl:MWCNTs/ISM	0.32	0.31	2.97

**Table 4:** Selected analytical parameters obtained for the tested electrodes.

Electrode	Slope, mV dec^−1^		Linear Range, mol L^−1^		Limit of Detection, mol L^−1^		Long-Term Stability E^0^ ± SD, mV
	1. Week	2. Month	1. Week	2. Month	1. Week	2. Month	
GCE/ISM	−59.7	−59.6	5 × 10^−5^–1 × 10^−1^	1 × 10^−4^–1 × 10^−1^	6.3 × 10^−6^	1.9 × 10^−5^	205.0 ± 56
GCE/PANINFs-Cl/ISM	−59.6	−60.2	5 × 10^−6^–1 × 10^−1^	1 × 10^−5^–1 × 10^−1^	2.6 × 10^−6^	6.5 × 10^−6^	69.0 ± 8.5
GCE/MWCNTs/ISM	−60.3	−60.2	5 × 10^−6^–1 × 10^−1^	5 × 10^−6^–1 × 10^−1^	2.8 × 10^−6^	4.8 × 10^−6^	225.2 ± 6.3
GCE/(1:2)PANINFs-Cl:MWCNTs/ISM	−61.1	−60.1	5 × 10^−6^–1 × 10^−1^	5 × 10^−6^–1 × 10^−1^	2.7 × 10^−6^	4.8 × 10^−6^	126.4 ± 6.1
GCE/(1:1)PANINFs-Cl:MWCNTs/ISM	−61.2	−60.5	5 × 10^−6^–1 × 10^−1^	5 × 10^−6^–1 × 10^−1^	2.7 × 10^−6^	3.8 × 10^−6^	105.7 ± 3.5
GCE/(2:1)PANINFs-Cl:MWCNTs/ISM	−61.3	−61.1	5 × 10^−6^–1 × 10^−1^	5 × 10^−6^–1 × 10^−1^	2.3 × 10^−6^	3.6 × 10^−6^	89.5 ± 1.8

**Table 5:** Reversibility and short-term stability of the electrode potential determined in NaCl solutions with a concentration of 10^−4^ and 10^−3^ mol L^−1^. Mean values and standard deviation obtained for 5 results.

Electrode	10^−4^ mol L^−1^		10^−3^ mol L^−1^		Potential Drift, mV h^−1^
	Mean, mV	SD, mV	Mean, mV	SD, mV	
GCE/ISM	438.83	13.24	402.58	7.85	7.60
GCE/PANINFs-Cl/ISM	310.78	4.21	250.12	2.78	0.56
GCE/MWCNTs/ISM	470.10	3.82	405.58	2.26	0.08
GCE/(1:2)PANINFs-Cl:MWCNTs/ISM	370.48	3.17	310.81	2.39	0.35
GCE/(1:1)PANINFs-Cl:MWCNTs/ISM	350.39	2.19	290.98	1.87	0.09
GCE/(2:1)PANINFs-Cl:MWCNTs/ISM	335.26	1.42	270.80	0.71	0.03

**Table 6:** Determination of chlorides in water samples by direct potentiometry and comparison with the classic Mohr’s method.

Sample	Chloride Content Found by Proposed ISE, mmol L^−1^	Chloride Content Found by Classic Mohr’s Method, mmol L^−1^
Tap water	0.737 ± 0.016	0.745
Mineral water	0.311 ± 0.018	0.302
River water	1.07 ± 0.026	1.01

**Table 7:** Comparison of the parameters for the tested chloride electrodes and commercially available electrodes.

Name of Electrode	Producer	Ion-Sensitive Membrane Type	Slope, mV dec^−1^	Linear Range, mol L^−1^	Detection Limit, mol L^−1^	Interfering Ions with logK ≥ −2	Response Time, s	pH Range	Ref.
Chloride ISE GCE/(2:1)PANINFs-Cl:MWCNTs/ISM	-	PVC	−61.3	5 × 10^−6^–1 × 10^−1^	2.56 × 10^−6^	Br-	<10	4–9	This work
Chloride electrode ECl-01	ELMETRON	Polycrystalline	−56 ± 3	5 × 10^−5^–1	-	Br^−^, S_2_O_3_^2−^, I^−^, S^2-^	30–60	2–11	[45]
Intellical ISECL181 chloride ISE (Cl⁻)	HACH	Solid-state crystal membrane	-	3 × 10^−6^–1	-	-	-	-	[46]
ISE Hanna HI 4107	MERA	Semiconductor, combined	-	1 × 10^−5^–1	-	-	-	2–11	[47]
Chloride ISE	VERNIER	-	−56 ± 3	3 × 10^−5^–1	-	CN^–^, Br^–^, I^–^, OH^–^, S^2–^, NH_3_	-	2–12	[48]
Orion™ Chloride Electrode 9417SC	THERMOFISHER	-	-	1 × 10^−5^–1	-	-	-	-	[49]

## Data Availability

Not applicable.
